# MINEs: open access databases of computationally predicted enzyme promiscuity products for untargeted metabolomics

**DOI:** 10.1186/s13321-015-0087-1

**Published:** 2015-08-28

**Authors:** James G Jeffryes, Ricardo L Colastani, Mona Elbadawi-Sidhu, Tobias Kind, Thomas D Niehaus, Linda J Broadbelt, Andrew D Hanson, Oliver Fiehn, Keith E J Tyo, Christopher S Henry

**Affiliations:** Department of Chemical and Biological Engineering, Northwestern University, Evanston, IL USA; Mathematics and Computer Science Division, Argonne National Laboratory, Argonne, IL USA; West Coast Metabolomics Center, University of California, Davis, CA USA; Horticultural Sciences Department, University of Florida, Gainesville, FL USA; Biochemistry Department, King Abdulaziz University, Jeddah, Saudi Arabia

**Keywords:** Enzyme promiscuity, Untargeted metabolomics, Liquid chromatography–mass spectrometry, Metabolite identification

## Abstract

**Background:**

In spite of its great promise, metabolomics has proven difficult to execute in an untargeted and generalizable manner. Liquid chromatography–mass spectrometry (LC–MS) has made it possible to gather data on thousands of cellular metabolites. However, matching metabolites to their spectral features continues to be a bottleneck, meaning that much of the collected information remains uninterpreted and that new metabolites are seldom discovered in untargeted studies. These challenges require new approaches that consider compounds beyond those available in curated biochemistry databases.

**Description:**

Here we present Metabolic In silico Network Expansions (MINEs), an extension of known metabolite databases to include molecules that have not been observed, but are likely to occur based on known metabolites and common biochemical reactions. We utilize an algorithm called the Biochemical Network Integrated Computational Explorer (BNICE) and expert-curated reaction rules based on the Enzyme Commission classification system to propose the novel chemical structures and reactions that comprise MINE databases. Starting from the Kyoto Encyclopedia of Genes and Genomes (KEGG) COMPOUND database, the MINE contains over 571,000 compounds, of which 93% are not present in the PubChem database. However, these MINE compounds have on average higher structural similarity to natural products than compounds from KEGG or PubChem. MINE databases were able to propose annotations for 98.6% of a set of 667 MassBank spectra, 14% more than KEGG alone and equivalent to PubChem while returning far fewer candidates per spectra than PubChem (46 vs. 1715 median candidates). Application of MINEs to LC–MS accurate mass data enabled the identity of an unknown peak to be confidently predicted.

**Conclusions:**

MINE databases are freely accessible for non-commercial use via user-friendly web-tools at http://minedatabase.mcs.anl.gov and developer-friendly APIs. MINEs improve metabolomics peak identification as compared to general chemical databases whose results include irrelevant synthetic compounds. Furthermore, MINEs complement and expand on previous in silico generated compound databases that focus on human metabolism. We are actively developing the database; future versions of this resource will incorporate transformation rules for spontaneous chemical reactions and more advanced filtering and prioritization of candidate structures.

**Electronic supplementary material:**

The online version of this article (doi:10.1186/s13321-015-0087-1) contains supplementary material, which is available to authorized users.

**Graphical abstract:**
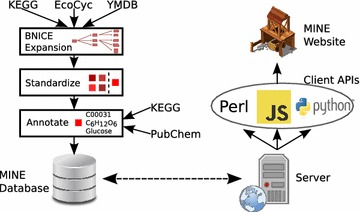
MINE database construction and access methods. The process of constructing a MINE database from the curated source databases is depicted on the left. The methods for accessing the database are shown on the right.

## Background

Metabolomics, the study of the population of small molecules in a cell, has drawn intense interest in fields from medicine to synthetic biology because it can provide a fine-grain representation of cellular state and activity [1–4]. Of particular interest is untargeted metabolomics, which seeks to measure as much of the metabolome as possible by limiting methodological detection bias. The dominant analysis technique for untargeted metabolomics is chromatography coupled with mass spectrometry (MS) but this method is hindered by a large number of unknown peaks [5] and the limited number of reference spectra available to identify the peaks [6]. A number of tools have been developed to propose structural matches for unannotated peaks [7–11] but in practice these tools either return too many candidates when drawing from large chemical databases such as PubChem [12] or miss compounds not yet present in curated biochemical database [13, 14].This has the effect of locking untargeted metabolomics in a unfortunate paradox: compounds that are not present in biochemical databases are not identified and in the absence of experimental identification, new compounds cannot be added to databases [15].

There is a growing consensus that many enzymes mediate undocumented side-reactions (known as promiscuous activities) as a result of exposure to diverse cellular metabolites [16, 17]. These activities may explain unannotated peaks in metabolomics datasets [18, 19] but are difficult to detect as they may be overshadowed by a known function [20] or be dependent on intracellular conditions [21]. Predicting novel chemical reactions based on broad enzyme specificity has been utilized by a number of tools for the prediction of new biochemical pathways [22–24]. Recently, this technique has also been used to expand structure databases for metabolomics by the MyCompoundID tool [25] the In Vivo/In Silico Metabolites Database (IIMDB) [15], LipidHome [26] and others [27, 28].

Here we present Metabolic In silico Network Expansions (MINEs) that utilize the Biochemical Network Integrated Network Explorer (BNICE) [29, 30] to expand on general biochemical databases as well as organism-specific databases for *Escherichia coli* and yeast. The focus on endogenously present and organism-specific metabolites has been cited as critical to improving the confidence of compound matches [5] and thus we complement existing resources that focus on human metabolism. In principle, these predictions could also be made using Reaction Difference Matching (RDM) [23], machine learning methods [31, 32], or other rule-based methods such as ChemAxon’s Metabolizer. Each of these approaches has their benefits; the output really depends on the quality and coverage of the reaction rules used in the analysis. We selected BNICE because we have a set of BNICE reaction rules that have been demonstrated to reproduce a large fraction of known biochemical reactions [24], as well as to predict enzyme reactions that were subsequently verified experimentally [33]. Importantly, we also have the right to re-distribute BNICE output. No license is required for academic users to access the website or APIs and all BNICE predicted compounds are available for download in SDF format from the website.

## Construction and content

**Fig. 1:**
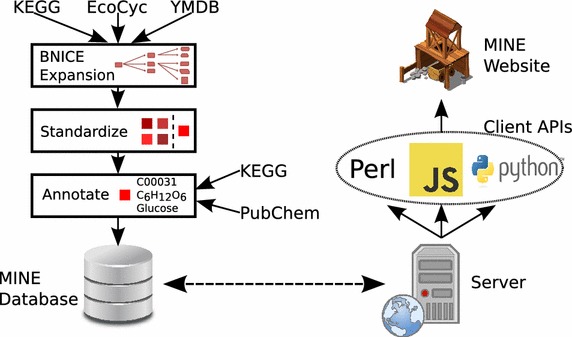
MINE database construction and access methods. The process of constructing a MINE database from the curated source databases is depicted on the *left*. The methods for accessing the database are shown on the *right*.

Construction of MINE databases follows the steps depicted in Fig. 1: BNICE expansion, Standardization and Annotation. The standardization and annotation procedure was guided by previous databases that combine reaction and compound data from various sources [34, 35].

Compound information was obtained from the Kyoto Encyclopedia of Genes and Genomes (KEGG) (Release 68.0) [36], the Yeast Metabolome Database (YMDB) (Version 1.0) [37] and EcoCyc (Version 17.0) [38]. Generalized (containing R groups), inorganic compounds, and disconnected fragments were removed using the Pybel toolkit [39]. Generalized structures are of very limited utility, as they cannot be assigned an accurate mass or represented in a canonical form. Where possible, we encourage developers to avoid ambiguity by enumerating all possible structures in their databases. Additionally, biochemical databases often contain numerous duplicate compounds [40] and these were identified by Standard InChIKey [41] comparison and removed for computational efficiency.

**Fig. 2:**
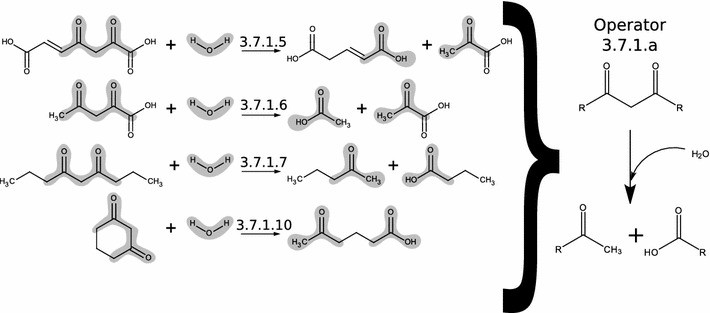
Generalizing a BNICE reaction rule from known biochemical reactions. The common motif of the hydrolysis of the 1,3-diketone is *shaded for emphasis*.

The BNICE framework has previously been used to explore alternate biosynthetic and xenodegradation pathways through the iterative application of generalized reaction rules. Unlike some approaches that model only a specific class of chemistry (e.g. cytochrome P450 metabolism) these reaction rules span the breadth of the Enzyme Commission (EC) classification system and have been hand curated by examining reactions at the third level of EC specificity. Figure 2 demonstrates the process of encoding the common reactive site motifs as well as the bonds that are broken or formed. 198 of these generalized chemical reaction rules were applied to all compounds in a given source database, resulting in a MINE database of predicted products and chemical reactions.

BNICE products may take a variety of tautomeric forms depending on the source structure and the nature of the operator applied. Therefore, products were processed with ChemAxon’s Standardizer & Structure Checker (JChem 6.0.4, 2013) to ensure canonical valences and placement of charge. Natural Product Likeness scores [42] and estimated logP values were calculated with a standalone Java ARchive (JAR) package and ChemAxon’s Calculator Plugins (JChem 6.0.4, 2013) respectively. Estimated Kováts Retention Indices were calculated using the NIST RI algorithm [43].

Compounds were matched to PubChem [44] and KEGG COMPOUND databases with the connectivity block of InChIKeys for annotation. Generated compounds are assigned identifiers based on hash of the canonical SMILES [45] for internal use and a numeric MINE ID for human readability. Finally, the exact mass and chemical fingerprints of structures were calculated with Pybel.

Compound and reaction data is stored as collections in a Mongo Database (v2.6.2). A compound entry contains the chemical formula, exact mass, InChIKey canonical SMILES [45], FP2 and FP4 fingerprints and lists of reactions in which the compound is predicted to participate as a reactant or product. A compound may also be annotated with additional information such as common names or database links if it matches a KEGG or PubChem entry. Reactions are uniquely identified by an ‘R’ followed by the SHA1 hash of the sorted chemical reaction. Reactions entries contain arrays of reactants and products as tuples of the stoichiometric coefficient and the compound ID as well as a list of the operators that predicted the reaction.

## Utility and discussion

### Database validation

Table 1 summarizes a few key statistics to compare MINEs to other commonly used databases. The most conservative metabolite-prediction database is IIMDB [15], which utilizes a combination of absolute and relative reasoning rules [46] based on human xenometabolism to constrain the size of the database. Two other methods using computationally-predicted metabolites, MyCompoundID [25] and Ridder et al.’s green tea metabolites [27], begin with much smaller metabolite starting sets than KEGG COMPOUND but utilize broader reaction rules and permit more sequential transformations. MINE operators specify reactant substructures but involve no relative likeliness calculations and therefore generate more compounds than IIMDB, but less than MyCompoundID. The relative increase between the starting metabolite set and the resulting MINE is dependent on the specific compounds present in the starting database. For example, YMDB contains more high-molecular-weight compounds than EcoCyc and thus contains more reaction sites and generates more derivatives. Like the IIMDB, the majority of compounds in MINE databases are not found in PubChem (when searching with the InChIKey connectivity block), which indicates MINEs are largely composed of novel structures. An analysis of the overlap in compounds represented in IIMDB was not performed due to licensing restrictions.

**Table 1:** Comparison of MINEs generated from various source databases and other databases containing computationally predicted metabolites

	Original database compounds	Final database compounds	Fold increase	Compounds found in PubChem (%)
KEGG MINE	13,307	571,368	43	6.99
EcoCyc MINE	1,832	54,719	30	11.27
YMDB MINE	1,978	100,755	51	7.26
IIMDB [15]	23,035	400,414	18	5.11
MyCompoundID [25] (2 generations)	8021	375,809 (10,583,901)	47 (1,320)	Unknown
Green Tea metabolites [27]	75	27,170	363	1.58

**Fig. 3:**
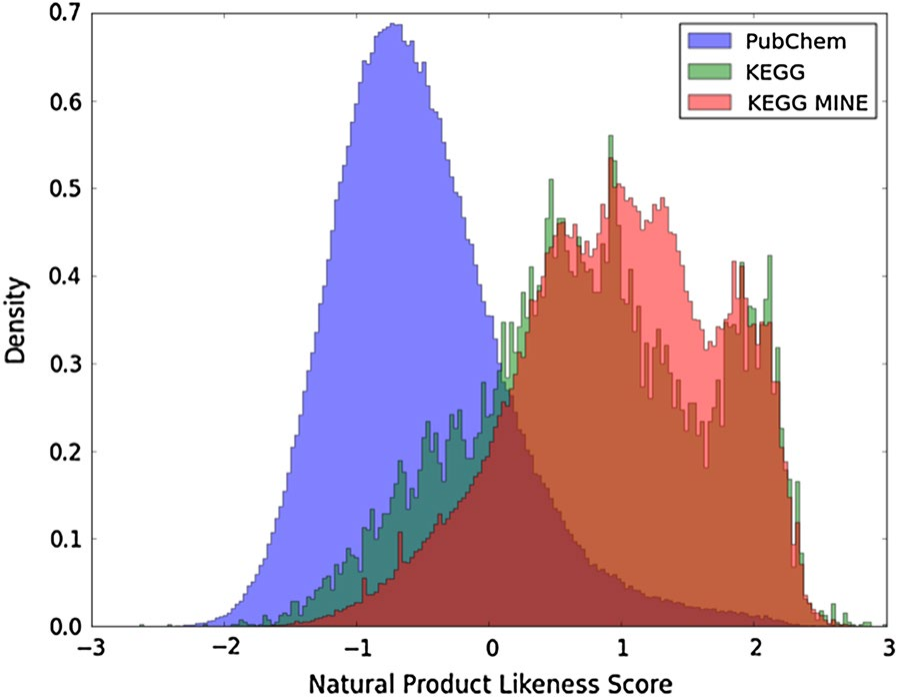
Histogram of Natural Product Likeness. This plot shows the distribution of Natural Product Likeness Scores for the KEGG Database (mean score 0.77), the KEGG MINE (mean score 0.98) and a random sample of 500,000 PubChem compounds (mean score −0.52). A more positive score indicates more natural atomic features.

Figure 3 displays the Natural Product (NP) Likeness scores [42] for 500,000 randomly sampled PubChem compounds, and the entirety of the KEGG COMPOUND and KEGG MINE databases. NP Likeness is calculated by scoring characteristic atomic signatures, which are present in the query molecule. Scores range from −3 to 3 with higher scores indicating a compound that contains more natural than synthetic structural features. Despite being a common source of candidate structures for annotating metabolomics data, the PubChem sample is clearly skewed towards synthetic compounds. In contrast, KEGG is primarily Natural Product-like compounds and the average KEGG MINE compound is even more so. This shift is due to the action of reaction rules in BNICE that mimic detoxification metabolism acting on the least natural compounds in KEGG and additional reactivity of operators with high NP likeness (see Additional file 1). This bias toward NP-like compounds makes it a preferable source for candidate structures for unknown pathway intermediates and peaks in untargeted experiments.

### Web interface description

**Fig. 4:**
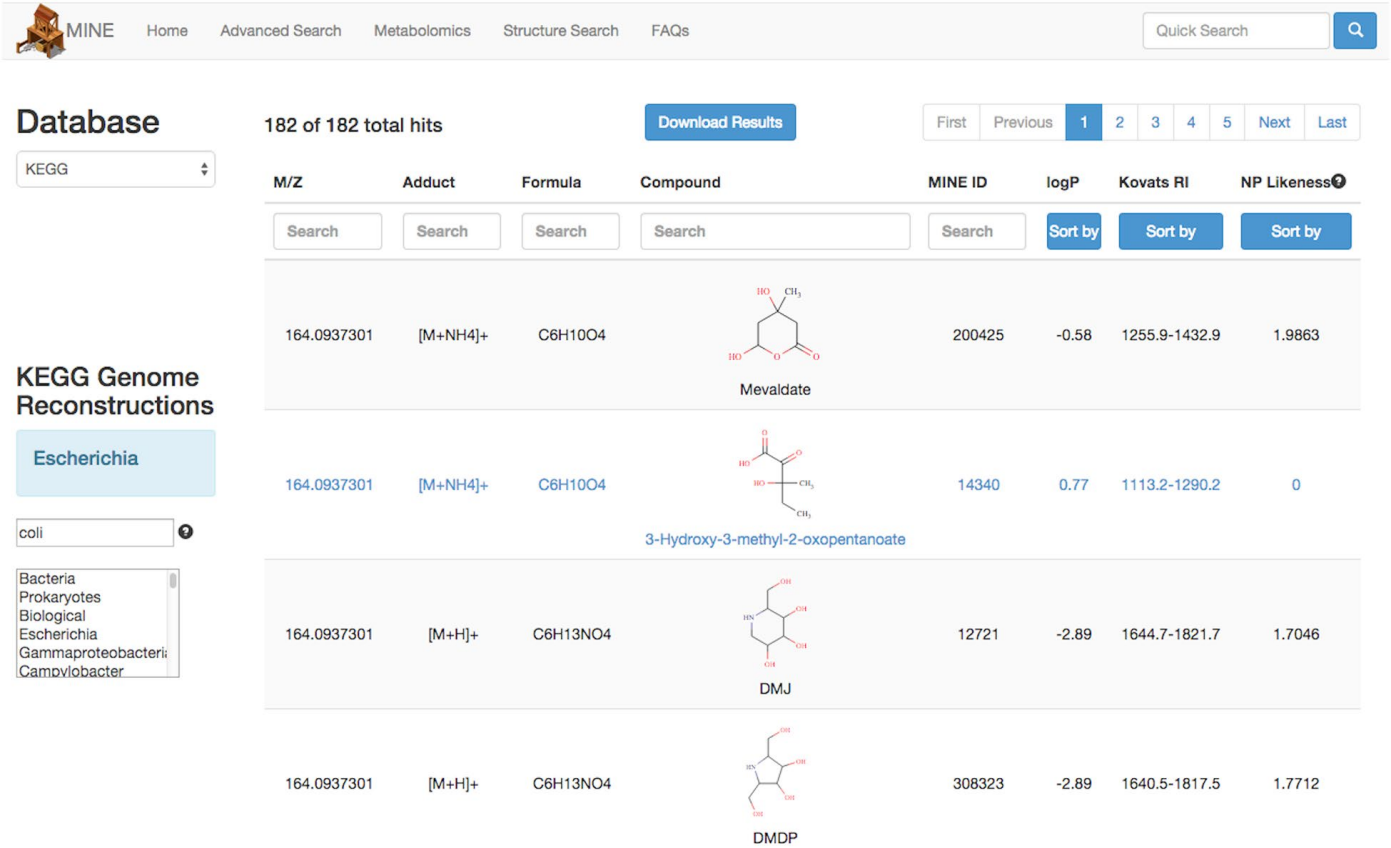
Screenshot of Metabolomics search results. This screenshot displays features of the metabolomics results including filtering by attributes and highlighting (*blue*) of compound present in a specified KEGG genome reconstruction.

The web interface for the MINE databases has been designed for a range of user needs such as (a) investigation of potential enzymatic transformations, (b) annotation of accurate masses and (c) chemical structure search. Users may access a compound of interest with a variety of identifiers such as InChI Keys, database IDs or common names, or with structure based tools like substructure and structural similarity searching. Compound pages display a set of name, pathway and enzyme annotations inferred from KEGG as well as the in silico predicted reactions that a compound may take part in as a reactant or product. Additionally, we provide a web interface for the annotation of accurate mass LC–MS data as shown in Fig. 4. This utility provides users a way to search for potential matches for a large number of mass-to-charge ratios and a color-coded interface that enables users to rapidly focus on the most probable putative identifications.

### Use case: annotation of accurate mass datasets

As a demonstration of the potential of MINEs for annotation of accurate mass data, a diverse test set of 667 unique compounds was compiled from MassBank [47]. The databases were searched by exact precursor mass to charge (*m*/*z*) ratio with 2 mDa precision and with [M+]^+^, [M+H]^+^, [M+Na]^+^, [M−H]^−^ and [M+CH_3_COO]^−^ adducts. The results of this validation are displayed in Table 2. Using KEGG as source database, structures were suggested for 84.5% of the *m*/*z*. The KEGG MINE database annotated an additional 14% of compounds while maintaining a similar accuracy to the KEGG annotations. PubChem annotates a comparable number of these known compounds to the KEGG MINE but does so at the expense of returning a bin of candidates that is two orders of magnitude larger than the MINE. While the MINE database has a higher median number of structures per peak than the KEGG database, the number remains feasible to examine manually. The web interface facilitates this process by distinguishing compounds that are present in user specified KEGG genome reconstructions from those generated by computational means, hence allowing users to consider the most probable isomers first. Additionally, users may restrict structures to a range of partition coefficients or Kováts retention index values. Candidate structures can then be downloaded as a Microsoft Excel compatible CSV file for further review.

**Table 2:** Annotation of MassBank data

	KEGG	KEGG MINE	PubChem
Features annotated	84.5%	98.6%	98.5%
Correct annotation present	68.6%	66.8%	89.8%
Median # of candidates	3	46	1714.5

Finally, to demonstrate the practical utility of MINE databases, we utilized the EcoCyc MINE to annotate untargeted metabolomics data from an *E. coli* knockout study analyzed by LC–MS. The protocols for sample extraction, data acquisition and post processing are available in the supplementary information. 493 distinct exact MS features were extracted, 30 of which were identified following a traditional annotation workflow using NIST MSPepsearch (see Additional file 2); in contrast, the EcoCyc MINE database proposed candidates for 132 of the accurate masses when searching with 5 mDa precision and with [M+]^+^, [M+H]^+^, [M+Na]^+^ adducts. The resulting MINE candidates were consistent with 93% of the NIST MSPepsearch results.

**Fig. 5:**
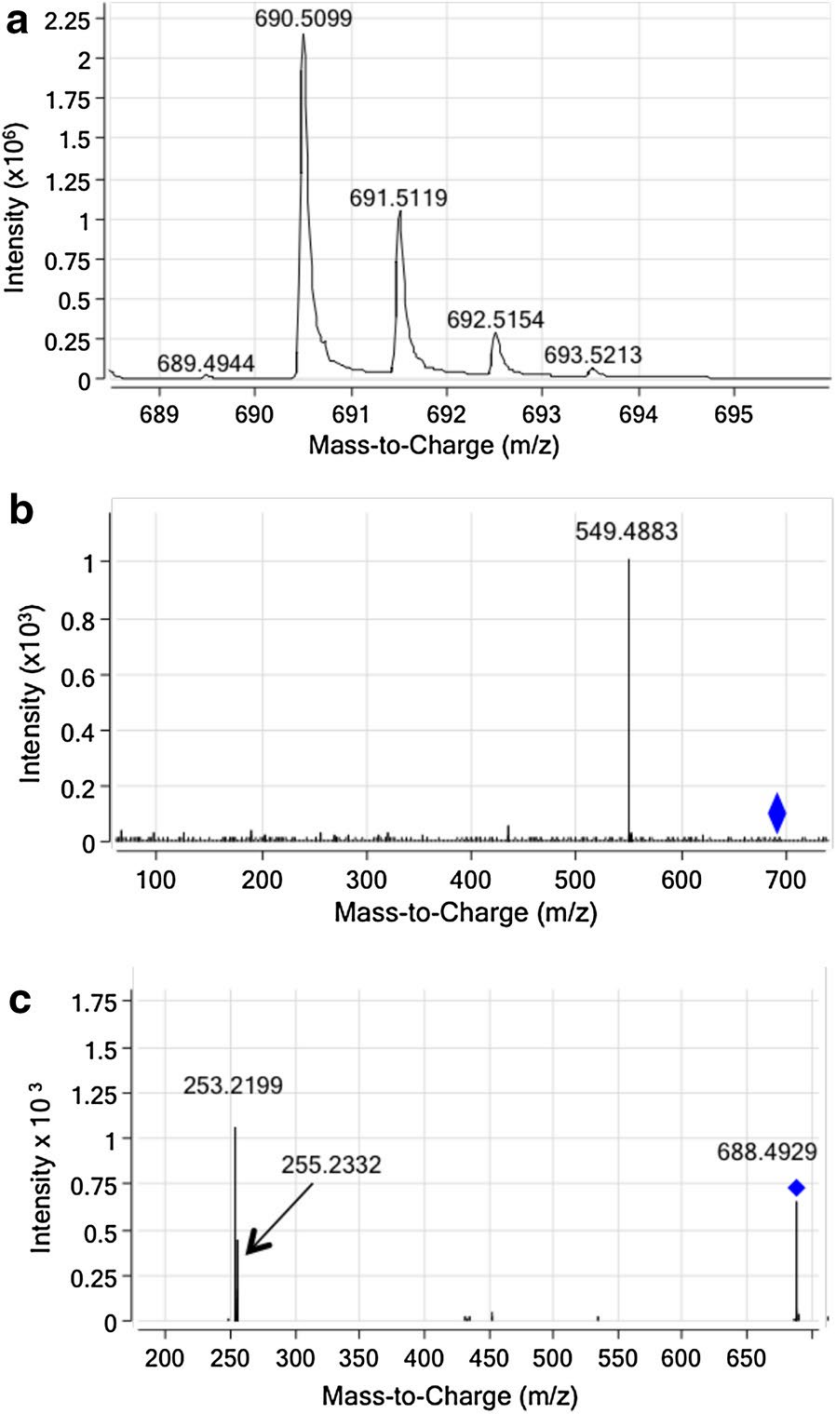
Positive MS spectrum (**a**), positive MS/MS spectrum (**b**) and negative MS/MS spectrum (**c**). The positive MS spectrum provides the mass of the precursor ion [M+H]^+^ = 690.5099 Da and its isotopic abundance pattern. The prominent ion in the positive MS/MS spectrum corresponds to the neutral loss of the phosphoethanolamine head group. The negative MS/MS spectrum shows the molecular ion [M−H]^−^ as well as a pair of ions corresponding to the (16:0) and (16:1) side chains.

Of these 132 features, 79 matched at least one of the metabolites proposed in the MINEs by the BNICE method. We selected one of these features, which also exhibited statistically significant variation in peak height across our experimental samples, for further study. The EcoCyc MINE database returned one potential hit for this metabolite, a phosphoethanolamine (PE) lipid that we were not able to identify with our traditional workflow. LipidBlast [11] was used to confirm that the MS–MS fragmentation pattern, presented in Fig. 5, is consistent with PE (32:1), more specifically, PE (16:0/16:1), which is also present as a predicted but unidentified lipid in the LipidHome database [26]. Detection and verification of novel metabolites is ongoing but beyond the scope of this article.

### Further development

In addition to the existing web tools, the underlying MINE databases are accessible through free, developer-friendly APIs. Clients are available for integration into Python, Perl and JavaScript frameworks at https://github.com/JamesJeffryes/MINE-API. This API allows the databases to be integrated into existing candidate ranking algorithms and pipelines. Future versions of these databases will incorporate transformation rules for spontaneous chemical reactions of metabolites, and improved filtering and prioritization of candidate structures.

In addition to expanding the scope for the metabolome, the MINE framework also offers a pipeline for illuminating the synthesis and degradation of poorly annotated secondary metabolites. While applied very broadly to nearly all of metabolism in this study, BNICE expansions may be focused on a region of interest in the metabolic network by adjusting the starting compounds and permissible transformations in a manner similar to that recently demonstrated by Ridder et al. [27]. These targeted MINEs will integrate the generation of plausible pathways by BNICE with the tools to detect the presence of predicted pathway intermediates with accurate mass spectrometry thereby accelerating the process of proposing and evaluating hypothetical enzymatic synthesis routes for a number of compounds of interest.

## Conclusions

Here we have presented Metabolic In silico Network Expansions (MINEs) that utilizes generalized biochemical transformations to propose structures for use in untargeted metabolomics. The resulting compounds are rarely found in PubChem but are structurally similar to natural products. We have demonstrated the utility of these databases for proposing correct metabolite structures that stymied a standard annotation workflow. MINE data are accessible without licensing restrictions for non-commercial users through a user-friendly web interface and API for developers in several common scripting languages.

## Availability and requirements

MINE databases are freely accessible at: http://minedatabase.mcs.anl.gov and API clients are available at https://github.com/JamesJeffryes/MINE-API. There are no restrictions for Academic Use. Commercial users must obtain a license from Pathway Solutions Inc. (www.pathway.jp) and explicit permission from the authors.

## Additional files

Additional file 1: Transformations of High and low Natural Product Likeness compounds. Additional file 2: Sample extraction, LC–MS data collection and processing protocols and repository access.

## Abbreviations

MINE: Metabolic In Silico Network Expansions
NP: natural products
LC–MS: liquid chromatography–mass spectrometry
BNICE: Biochemical Network Integrated Computational Explorer
KEGG: Kyoto Encyclopedia of Genes and Genomes
IUPAC: International Union of Pure and Applied Chemistry
InChI: IUPAC International Chemical Identifier
API: Application Programing Interface

## References

[1] Patti GJ, Yanes O, Siuzdak G (2012). Innovation: metabolomics: the apogee of the omics trilogy. Nat Rev Mol Cell Biol.

[2] Dromms R, Styczynski M (2012). Systematic applications of metabolomics in metabolic engineering. Metabolites.

[3] Roux A, Lison D, Junot C, Heilier J-F (2011). Applications of liquid chromatography coupled to mass spectrometry-based metabolomics in clinical chemistry and toxicology: a review. Clin Biochem.

[4] Guertin KA, Moore SC, Sampson JN, Huang W-Y, Xiao Q, Stolzenberg-Solomon RZ (2014) Metabolomics in nutritional epidemiology: identifying metabolites associated with diet and quantifying their potential to uncover diet-disease relations in populations. Am J Clin Nutr ajcn.113.078758 10.3945/ajcn.113.078758 PMC4144099 24740205

[5] Scalbert A, Brennan L, Fiehn O, Hankemeier T, Kristal BS, van Ommen B (2009). Mass-spectrometry-based metabolomics: limitations and recommendations for future progress with particular focus on nutrition research. Metabolomics.

[6] Stein S (2012). Mass spectral reference libraries: an ever-expanding resource for chemical identification. Anal Chem.

[7] Heinonen M, Shen H, Zamboni N, Rousu J (2012). Metabolite identification and molecular fingerprint prediction through machine learning. Bioinformatics.

[8] Menikarachchi LC, Cawley S, Hill DW, Hall LM, Hall L, Lai S et al (2012) MolFind: a software package enabling HPLC/MS-based identification of unknown chemical structures. Anal Chem 84:9388–9394 10.1021/ac302048x PMC3523192 23039714

[9] Wang Y, Kora G, Bowen B, Pan C (2014) MIDAS: a database-searching algorithm for metabolite identification in metabolomics. Anal Chem 86:9496–9503 10.1021/ac5014783 25157598

[10] Wolf S, Schmidt S, Müller-Hannemann M, Neumann S (2010). In silico fragmentation for computer assisted identification of metabolite mass spectra. BMC Bioinform.

[11] Kind T, Liu K-H, Lee DY, DeFelice B, Meissen JK, Fiehn O (2013). LipidBlast in silico tandem mass spectrometry database for lipid identification. Nat Methods.

[12] Schymanski E, Neumann S (2013). CASMI: and the winner is…. Metabolites.

[13] Shen H, Zamboni N, Heinonen M, Rousu J (2013). Metabolite identification through machine learning—tackling CASMI challenge using FingerID. Metabolites.

[14] Matsuda F (2014). Rethinking mass spectrometry-based small molecule identification strategies in metabolomics. Mass Spectrom.

[15] Menikarachchi LC, Hill DW, Hamdalla MA, Mandoiu II, Grant DF (2013). In silico enzymatic synthesis of a 400,000 compound biochemical database for nontargeted metabolomics. J Chem Inf Model.

[16] Nam H, Lewis NE, Lerman JA, Lee D-H, Chang RL, Kim D (2012). Network context and selection in the evolution to enzyme specificity. Science.

[17] Bar-Even A, Noor E, Savir Y, Liebermeister W, Davidi D, Tawfik DS et al (2011) The moderately efficient enzyme: evolutionary and physicochemical trends shaping enzyme parameters. Biochemistry 50:4402–4410 10.1021/bi2002289 21506553

[18] Weng J-K, Philippe RN, Noel JP (2012). The rise of chemodiversity in plants. Science.

[19] Fiehn O, Barupal DK, Kind T (2011). Extending biochemical databases by metabolomic surveys. J Biol Chem.

[20] O’Brien P, Herschlag D (1999) Catalytic promiscuity and the evolution of new enzymatic activities. Chem Biol 6:R91–R105 10.1016/S1074-5521(99)80033-7 10099128

[21] Sánchez-Moreno I, Iturrate L, Martín-Hoyos R, Jimeno ML, Mena M, Bastida A (2009). From kinase to cyclase: an unusual example of catalytic promiscuity modulated by metal switching. Chem Biochem.

[22] Gao J, Ellis LBM, Wackett LP (2011). The University of Minnesota Pathway Prediction System: multi-level prediction and visualization. Nucleic Acids Res.

[23] Moriya Y, Shigemizu D, Hattori M, Tokimatsu T, Kotera M, Goto S (2010). PathPred: an enzyme-catalyzed metabolic pathway prediction server. Nucleic Acids Res.

[24] Henry CS, Broadbelt LJ, Hatzimanikatis V (2010). Discovery and analysis of novel metabolic pathways for the biosynthesis of industrial chemicals: 3-hydroxypropanoate. Biotechnol Bioeng.

[25] Li L, Li R, Zhou J, Zuniga A, Stanislaus AE, Wu Y (2013). MyCompoundID: using an evidence-based metabolome library for metabolite identification. Anal Chem.

[26] Foster JM, Moreno P, Fabregat A, Hermjakob H, Steinbeck C, Apweiler R (2013). LipidHome: a database of theoretical lipids optimized for high throughput mass spectrometry lipidomics. PLoS One.

[27] Ridder L, van der Hooft JJJ, Verhoeven S, De Vos RCH, Vervoort J, Bino RJ (2014) In silico prediction and automatic LC–MS n annotation of green tea metabolites in urine. Anal Chem 140411210700006 10.1021/ac403875b 24779709

[28] Morreel K, Saeys Y, Dima O, Lu F, Van de Peer Y, Vanholme R et al (2014) Systematic structural characterization of metabolites in arabidopsis via candidate substrate-product pair networks. Plant Cell 26:tpc.113.122242 10.1105/tpc.113.122242 PMC4001402 24685999

[29] González-Lergier J, Broadbelt LJ, Hatzimanikatis V (2005). Theoretical considerations and computational analysis of the complexity in polyketide synthesis pathways. J Am Chem Soc.

[30] Henry CS, Jankowski MD, Broadbelt LJ, Hatzimanikatis V (2006). Genome-scale thermodynamic analysis of *Escherichia coli* metabolism. Biophys J.

[31] Mu F, Unkefer CJ, Unkefer PJ, Hlavacek WS (2011). Prediction of metabolic reactions based on atomic and molecular properties of small-molecule compounds. Bioinformatics.

[32] De Groot MJL, Van Berlo RJP, Van Winden WA, Verheijen PJT, Reinders MJT, De Ridder D (2009). Metabolite and reaction inference based on enzyme specificities. Bioinformatics.

[33] Frelin O, Huang L, Hasnain G, Jeffryes JG, Ziemak MJ, Rocca JR (2015). A directed-overflow and damage-control *N*-glycosidase in riboflavin biosynthesis. Biochem J.

[34] Kumar A, Suthers PF, Maranas CD (2012). MetRxn: a knowledgebase of metabolites and reactions spanning metabolic models and databases. BMC Bioinform.

[35] Lang M, Stelzer M, Schomburg D (2011). BKM-react, an integrated biochemical reaction database. BMC Biochem.

[36] Kanehisa M, Goto S, Sato Y, Kawashima M, Furumichi M, Tanabe M (2014). Data, information, knowledge and principle: back to metabolism in KEGG. Nucleic Acids Res.

[37] Jewison T, Knox C, Neveu V, Djoumbou Y, Guo AC, Lee J (2012). YMDB: the yeast metabolome database. Nucleic Acids Res.

[38] Keseler IM, Mackie A, Peralta-Gil M, Santos-Zavaleta A, Gama-Castro S, Bonavides-Martínez C (2013). EcoCyc: fusing model organism databases with systems biology. Nucleic Acids Res.

[39] O’Boyle NM, Morley C, Hutchison GR (2008). Pybel: a python wrapper for the OpenBabel cheminformatics toolkit. Chem Cent J.

[40] Altman T, Travers M, Kothari A, Caspi R, Karp PD (2013) A systematic comparison of the MetaCyc and KEGG pathway databases. BMC Bioinform 14:112 10.1186/1471-2105-14-112 PMC3665663 23530693

[41] Heller S, McNaught A, Stein S, Tchekhovskoi D, Pletnev I (2013). InChI: the worldwide chemical structure identifier standard. J Cheminform.

[42] Jayaseelan KV, Moreno P, Truszkowski A, Ertl P, Steinbeck C (2012). Natural product-likeness score revisited: an open-source, open-data implementation. BMC Bioinform.

[43] Stein SE, Babushok VI, Brown RL, Linstrom PJ (2007) Estimation of kovats retention indices using group contributions. J Chem Inf Model 47:975–980 10.1021/ci600548y 17367127

[44] Bolton E, Wang Y, Thiessen P, Bryant S (2008). PubChem: integrated platform of small molecules and biological activities. Annu Rep.

[45] Weininger D, Weininger A, Weininger JL (1989). SMILES. 2. Algorithm for generation of unique SMILES notation. J Chem Inf Model.

[46] Fenner K, Gao J, Kramer S, Ellis L, Wackett L (2008). Data-driven extraction of relative reasoning rules to limit combinatorial explosion in biodegradation pathway prediction. Bioinformatics.

[47] Horai H, Arita M, Kanaya S, Nihei Y, Ikeda T, Suwa K (2010). MassBank: a public repository for sharing mass spectral data for life sciences. J Mass Spectrom.

