# Supplementary material for: MINEs: open access databases of computationally predicted enzyme promiscuity products for untargeted metabolomics
Source: J Cheminform. 2015 Aug 28;7:44. doi: 10.1186/s13321-015-0087-1 (PMC4550642; doi:10.1186/s13321-015-0087-1)
Supplement: Additional file 1: — Transformations of High and low Natural Product Likeness compounds. [file 13321_2015_87_MOESM1_ESM.docx]

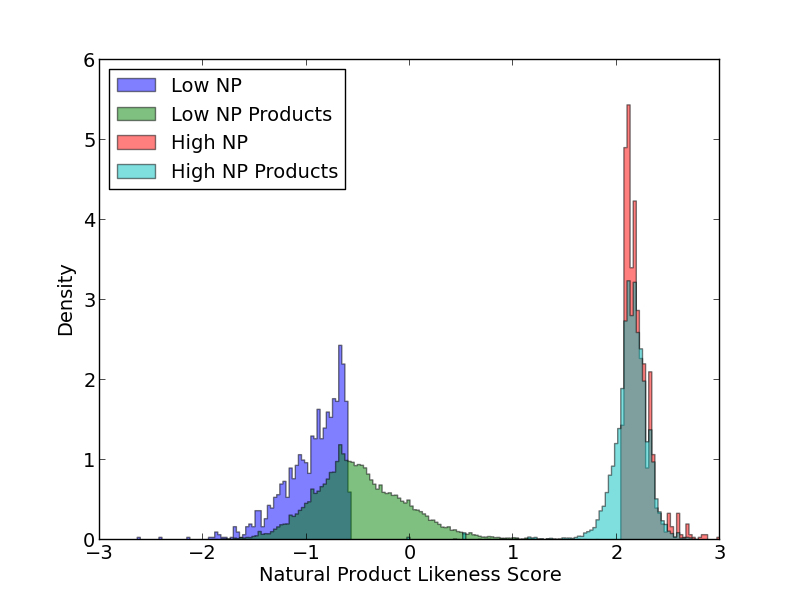


**Figure S1: Comparison of NP Likeness of Source and Product compounds of the KEGG MINE** This figure shows the distribution of NP Likeness scores for the 1000 KEGG compounds with the lowest NP Likeness(mean: -0.94) which result in 32,000 products (mean: -0.47) and contrast this with the 1000 KEGG compounds with the highest NP Likeness scores(mean: 2.20) which result in 49,000 products (mean: 2.13). Source and product distribution for the high NP Likeness compounds are very similar while the low NP likeness products clearly exhibit a shift towards more natural compounds.

**Table S1: Most Common Reaction Operators for High and Low NP Likeness compounds**

| **Low NP Likeness** | | **High NP Likeness** | |
| --- | --- | --- | --- |
| **Operator** | **Reactions** | **Operator** | **Reactions** |
| 1.14.13.a | 5681 | 1.14.13.a | 9651 |
| 1.3.-1.a | 4554 | 1.14.15.a | 7618 |
| 2.1.1.a | 4479 | 2.1.1.a | 4959 |
| 1.14.13.c | 3721 | 2.7.1.a | 3580 |
| 1.14.12.a | 3019 | 1.1.1.a | 3068 |
| 1.14.13.d | 956 | 2.8.2.a | 2626 |
| 3.8.1.a | 834 | 1.14.13.e | 2371 |
| 1.14.13.e | 769 | 1.3.1.a | 1625 |

This table displays the most common operators in the set of reactions starting from the 1000 KEGG compounds with the lowest and highest NP Likeness scores. While some are present in both lists due to their broad specificity (1.14.13.a & 2.1.1.a), the low NP Likeness compounds are enriched for monooxygenases (1.14.13.x), reductases (1.3.-1.a), C-halide hydrolases (3.8.1.x). All these actions are classically part of phase 1 xenometabolism.
