# Supplementary material for: MINEs: open access databases of computationally predicted enzyme promiscuity products for untargeted metabolomics
Source: J Cheminform. 2015 Aug 28;7:44. doi: 10.1186/s13321-015-0087-1 (PMC4550642; doi:10.1186/s13321-015-0087-1)
Supplement: Additional file 2: — Sample extraction, LC–MS data collection and processing protocols and repository access. [file 13321_2015_87_MOESM2_ESM.docx]

# Supplemental Methods

Six independent colonies from each of two *E. coli* lines (wild-type and *yjeF* knockout) were grown overnight in M9 minimal medium (0.2% glucose) and used to inoculate 2 mL of fresh medium to an optical density of 0.05 at 600 nm. Cultures were grown at 42°C with shaking for 5-6 h until optical density reached 1.3±0.1 at 600 nm, then an equivalent of 1 mL culture at an optical density of 2.0 was collected in 1.5-mL Eppendorf tubes by centrifugation and the pellet was frozen in liquid nitrogen. Samples were stored at -80°C.

Analysis by LC-MS was conducted on the supernatant from cell pellets extracted with 1 mL cold acetonitrile: isopropanol: water (3:3:2), dried overnight then resuspended with 30 *μ*L of acetonitrile:water (80:20) plus internal standards. Chromatography was performed on an Agilent 1290 Infinity LC System (Agilent Technologies, Santa Clara, CA) using a Waters Acquity 1.7 *μ*m BEH HILIC 150 x 2.1 mm UPLC column kept at 45^o^C. Mobile phase consisted of water with 10 mM ammonium formate and 0.125% formic acid (A) and 95:5 acetonitrile/water with 10 mM ammonium formate and 0.125% formic acid (B); flow rate was 0.4 mL/min throughout acquisition. The gradient method was 0 – 2min: 100% B, 2 – 7.7 min: 70% B, 7.7 – 9.5 min: 40% B, 9.5 – 10.25 min: 30% B, 10.25 – 12.75 min: 100% B, 12.75 – 16.75 min: 100% B. MS and MS/MS data were collected in positive and negative polarity with a 4 spectra/s scan rate and mass calibration was maintained by constant infusion of reference ions. Precursor ions were isolated with a narrow isolation width (~1.3 Da) and variable collision energy was applied based on precursor ion *m/z* for data-dependent MS/MS data generation.

Data processing was performed using the open-source software MZmine 2 [43] and the in-house MZmine post-processor (unpublished). Compound identification was based on accurate-mass and MS/MS matching in the NIST MSPepSearch GUI ([http://peptide.nist.gov/](http://peptide.nist.gov/)) with precursor ion and fragment peak tolerance of 0.05 *m/z* units using the NIST, Metlin, MassBank and LipidBlast libraries; additional MS/MS and retention time matching to in-house libraries was also performed.

Database API is implemented using US Department of Energy Biological Knowledge Base JSON Remote Procedure Call framework and is available at <https://bitbucket.org/jgj333/mine-server>. The MINE web application is built with AngularJS and available at <https://bitbucket.org/jgj333/mine-website>.
